# Supplementary material for: The HD-ZIP Gene Family in Watermelon: Genome-Wide Identification and Expression Analysis under Abiotic Stresses
Source: Genes (Basel). 2022 Nov 29;13(12):2242. doi: 10.3390/genes13122242 (PMC9777774; doi:10.3390/genes13122242)

Motif 1

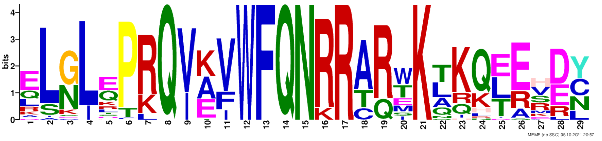

Motif 2

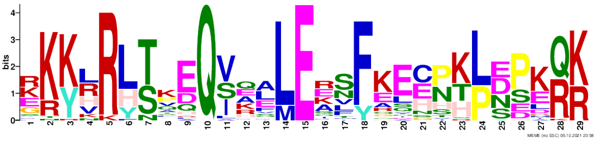

Motif 3

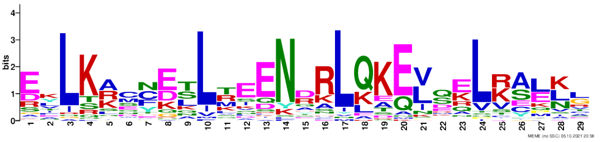

Motif 4

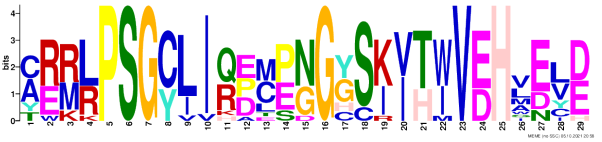

Motif 5

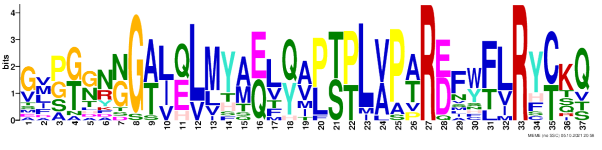

Motif 6

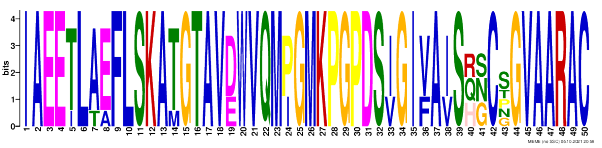

Motif 7

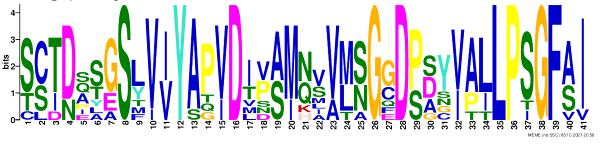

Motif 8

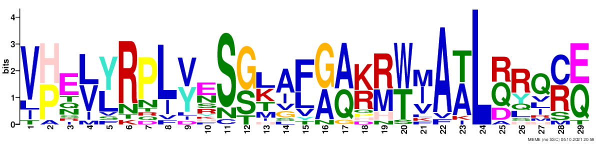

Motif 9

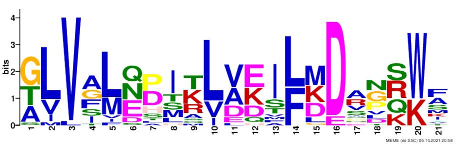

Motif 10

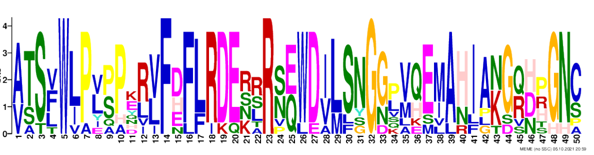

Motif 11

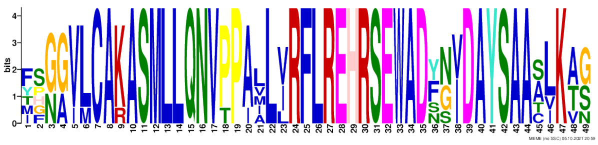

Motif 12

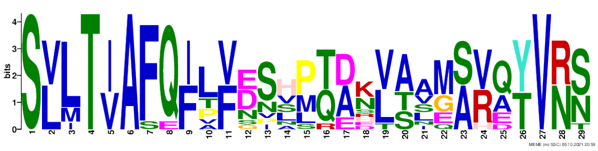

Motif 13

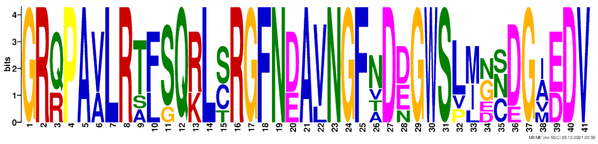

Motif 14

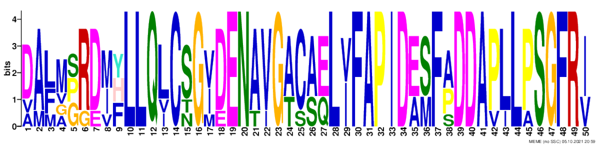

Motif 15

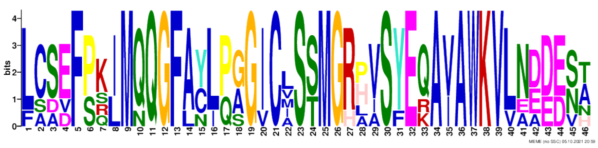

Motif 16

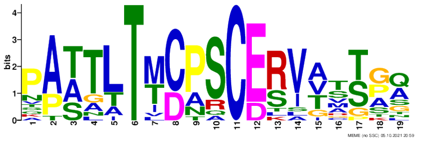

Motif 17

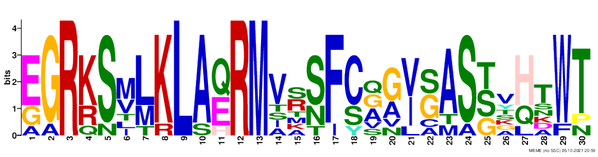

Motif 18

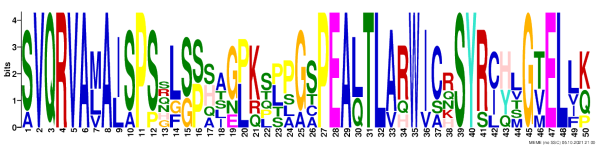

Motif 19

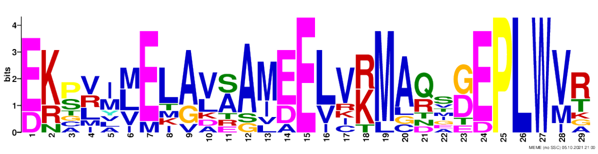

Motif 20

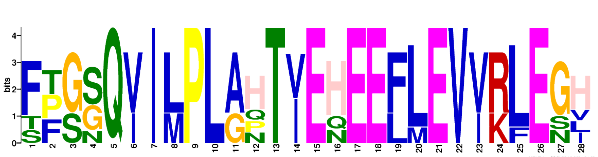

Supplement: Supplementary file 1 [file genes-13-02242-s001.zip › Figure S2.pdf]
