# Supplementary figures and images for: The HD-ZIP Gene Family in Watermelon: Genome-Wide Identification and Expression Analysis under Abiotic Stresses
Source: Genes (Basel). 2022 Nov 29;13(12):2242. doi: 10.3390/genes13122242 (PMC9777774; doi:10.3390/genes13122242)

**II****III****IV****I**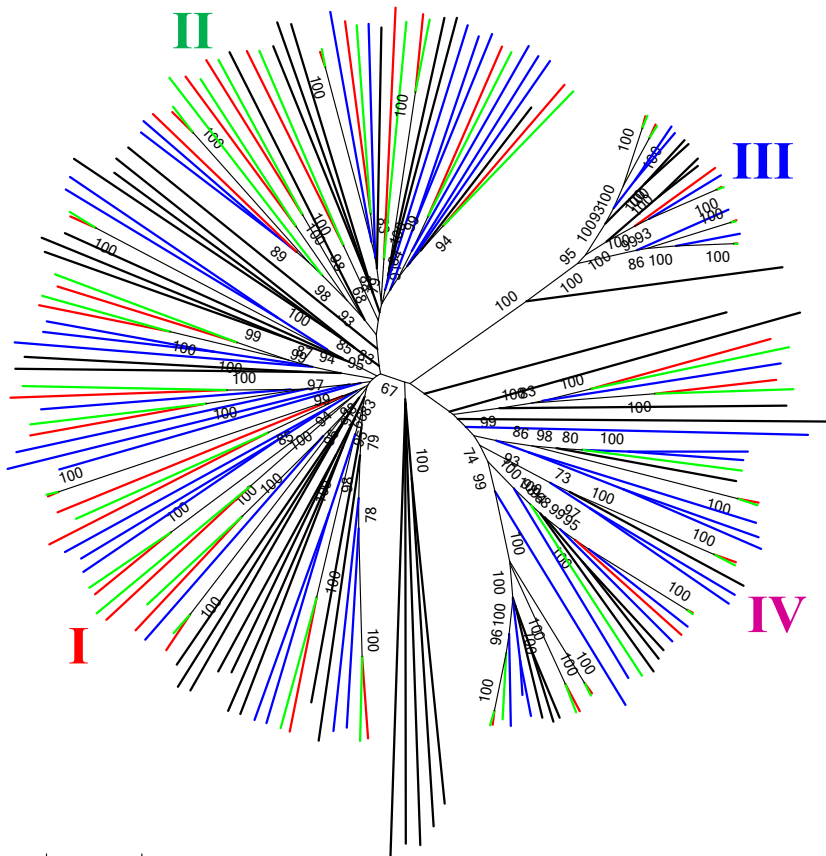

0.1

Supplement: Supplementary file 1 [file genes-13-02242-s001.zip › Figure S1.pdf]
